# Screening of gastric cancer diagnostic biomarkers in the homologous recombination signaling pathway and assessment of their clinical and radiomic correlations

**DOI:** 10.1002/cam4.70153

**Published:** 2024-08-29

**Authors:** Ahao Wu, Tengcheng Hu, Chao Lai, Qingwen Zeng, Lianghua Luo, Xufeng Shu, Pan Huang, Zhonghao Wang, Zongfeng Feng, Yanyan Zhu, Yi Cao, Zhengrong Li

**Affiliations:** ^1^ Department of Digestive Surgery, The First Affiliated Hospital, Jiangxi Medical College Nanchang University Nanchang Jiangxi China; ^2^ Medical Innovation Centre The First Affiliated Hospital of Nanchang University Nanchang Jiangxi China; ^3^ Department of Radiology The First Affiliated Hospital of Nanchang University Nanchang Jiangxi China; ^4^ Department of Digestive Surgery, Digestive Disease Hospital The Third Affiliated Hospital of Nanchang University Nanchang Jiangxi China

**Keywords:** gastric cancer, homologous recombination, RAD51D, radiomics, XRCC2

## Abstract

**Background:**

Homologous recombination plays a vital role in the occurrence and drug resistance of gastric cancer. This study aimed to screen new gastric cancer diagnostic biomarkers in the homologous recombination pathway and then used radiomic features to construct a prediction model of biomarker expression to guide the selection of chemotherapy regimens.

**Methods:**

Gastric cancer transcriptome data were downloaded from The Cancer Genome Atlas database. Machine learning methods were used to screen for diagnostic biomarkers of gastric cancer and validate them experimentally. Computed Tomography image data of gastric cancer patients and corresponding clinical data were downloaded from The Cancer Imaging Archive and our imaging centre, and then the Computed Tomography images were subjected to feature extraction, and biomarker expression prediction models were constructed to analyze the correlation between the biomarker radiomics scores and clinicopathological features.

**Results:**

We screened RAD51D and XRCC2 in the homologous recombination pathway as biomarkers for gastric cancer diagnosis by machine learning, and the expression of RAD51D and XRCC2 was significantly positively correlated with pathological T stage, N stage, and TNM stage. Homologous recombination pathway blockade inhibits gastric cancer cell proliferation, promotes apoptosis, and reduces the sensitivity of gastric cancer cells to chemotherapeutic drugs. Our predictive RAD51D and XRCC2 expression models were constructed using radiomics features, and all the models had high accuracy. In the external validation cohort, the predictive models still had decent accuracy. Moreover, the radiomics scores of RAD51D and XRCC2 were also significantly positively correlated with the pathologic T, N, and TNM stages.

**Conclusions:**

The gastric cancer diagnostic biomarkers RAD51D and XRCC2 that we screened can, to a certain extent, reflect the expression status of genes through radiomic characteristics, which is of certain significance in guiding the selection of chemotherapy regimens for gastric cancer patients.

## Graphical abstract

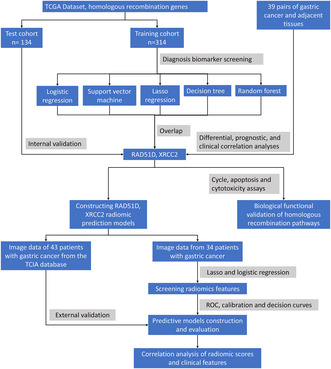

## 1 INTRODUCTION

Gastric cancer is one of the most common malignant tumors in the world, and its incidence rate is increasing annually.^1^ Early‐stage gastric cancer patients have excellent survival benefits after radical surgery,^2^, ^3^ while the overall survival of patients with advanced gastric cancer is still poor after comprehensive treatment.^4^, ^5^, ^6^ Therefore, early diagnosis of gastric cancer patients is crucial. *Helicobacter pylori* infection is recognized as an independent risk factor for gastric cancer, and the risk of gastric cancer is increased up to 6 times in those who are infected with *H. pylori*.^7^, ^8^ Recently, published studies have shown that mutations in cancer susceptibility genes in the homologous recombination pathway can increase the risk of gastric cancer by 1.68 times.^9^ People with both *H. pylori* infection and cancer susceptibility gene mutations in the homologous recombination pathway have an astonishing 22.45 times greater risk of gastric cancer. The synergistic effect between the two increases the risk of gastric cancer by 16 times. Therefore, homologous recombination plays a vital role in the occurrence of gastric cancer.

In recent years, the mechanisms of homologous recombination pathways in tumors have been increasingly studied.^10^ In lung cancer, homologous recombination repair deficiency leads to a high tumor mutation load and promotes the body to generate an immune response, thus exerting antitumor effects.^11^ In breast cancer, homologous recombination repair modulates tumor sensitivity to chemotherapeutic agents.^12^, ^13^ In colorectal cancer, homologous recombination defects were more common in the MSI‐H/dMMR group than in the MSS/pMMR group; patients with tumors in the MSS/pMMR group with homologous recombination defects exhibited longer overall survival.^14^ Patients with homologous recombination mutations who received platinum‐based chemotherapy had a significantly longer median overall survival than patients who did not receive platinum‐based chemotherapy.^15^ In gastric cancer, homologous recombination pathway activation improves the repair of DNA replication damage and resistance to platinum‐based chemotherapeutic agents.^16^, ^17^, ^18^, ^19^ In summary, variants in homologous recombination genes significantly increase the risk of gastric carcinogenesis; aberrant activation of homologous recombination pathways leads to resistance to platinum‐based chemotherapeutic agents in gastric cancer patients. Therefore, this study aimed to explore whether homologous recombination genes can serve as effective biomarkers for diagnosing gastric cancer, which is highly valuable for screening and early treatment.

Radiomics extracts many features from medical images and converts them into mineable data.^20^ Macroscopic images can reflect microscopic changes in tumors to a certain extent through radiomics feature extraction. In gastric cancer, a prediction model constructed by combining radiomics and deep learning can accurately predict the state of the tumor microenvironment.^21^ Currently, there are no published studies correlating CT radiomics features with gene expression. Therefore, in our study, we aimed to determine whether the radiomics features of Computed Tomography (CT) can also reflect the expression of diagnostic biomarkers. We further used radiomics features to construct a prediction model of biomarker expression that reflects the status of the homologous recombination pathway by predicting high or low expression of biomarkers and then determining the possibility of resistance to platinum‐based chemotherapy in gastric cancer patients to guide the selection of chemotherapy regimens for gastric cancer patients.

With the development of artificial intelligence, machine learning algorithms are widely used in the field of gastric cancer; and have considerable sensitivity and specificity.^22^, ^23^, ^24^ Here, we screened diagnostic biomarkers for homologous recombination signaling pathways through machine learning methods. The correlations between biomarkers and clinicopathological features, as well as the effects of the homologous recombination pathway on the gastric cancer cell cycle, apoptosis, and drug resistance, were verified. Finally, we evaluated the correlation between CT radiomics features and biomarker expression levels by constructing a prediction model for biomarker expression levels, calculating the radiomics scores of biomarkers, and analyzing the correlation between radiomics scores and clinicopathological features.

## 2 MATERIALS AND METHODS

### 2.1 Patients and samples

**FIGURE 1:**
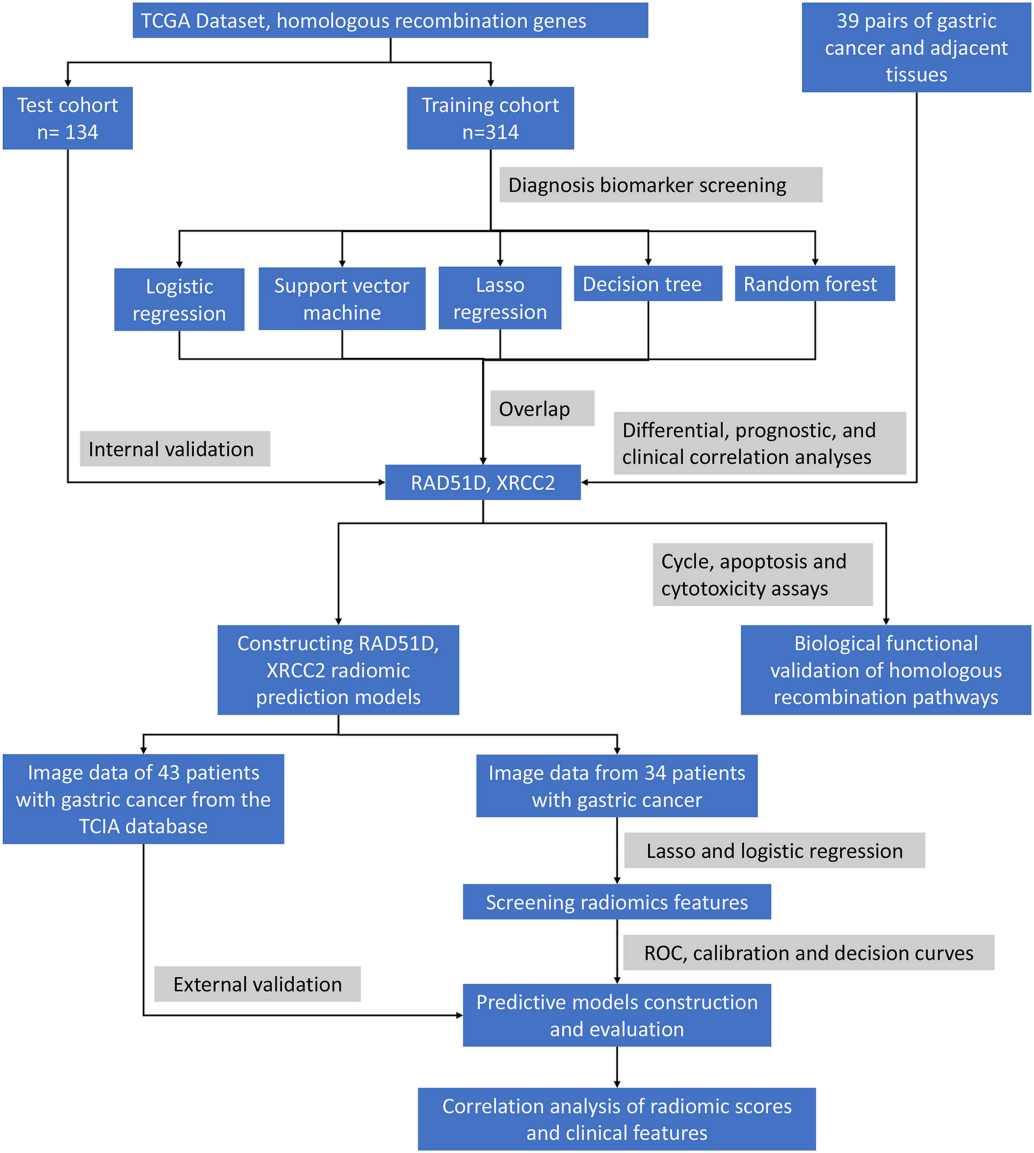
Flow of the study design.

Thirty‐nine pairs of gastric cancer tissues and adjacent normal mucosal tissues were collected from patients who underwent gastrectomy at the Gastrointestinal Surgery Department of the First Affiliated Hospital of Nanchang University from 2022 to 2023. None of the patients received radiotherapy or chemotherapy before surgery. All clinical specimens were frozen in liquid nitrogen or stored in a −80°C freezer immediately after collection, while the clinicopathological data of the corresponding patients were also collected. This study was approved by the Ethics Committee of the First Affiliated Hospital of Nanchang University [Ethics Committee Approval No: (2023) CDYFYYLK(09‐009)]. The specific research process is shown in Figure 1.

### 2.2 Screening of diagnostic biomarkers for gastric cancer

The transcriptome data of gastric cancer patients were downloaded from The Cancer Genome Atlas (TCGA) database (https://portal.gdc.cancer.gov/), which included data from 412 tumor tissue samples and 36 paracancerous tissue samples. The genes of homologous recombination pathways were obtained from the PathCards (https://pathcards.genecards.org/) website, and the expression data of homologous recombination genes in gastric cancer transcriptome data were extracted. The expression data of homologous recombination genes were divided randomly into a training group and a test group at a ratio of 7:3. The training group was used to screen feature genes, and the test group was used for internal validation. We employed learning methods such as logistic regression, least absolute shrinkage, and selection operator (LASSO) regression, support vector machine (SVM), and decision tree (DT) methods to screen for diagnostic biomarkers of homologous recombinant genes.

Download the package “e1071” in R Language. The SVM model was optimized with hyperparameters to determine the best cost and gamma values to construct the best SVM model. We used the “rpart” package in R language for decision analysis; the classification of the DT was from root to leaf. Statistics such as the Gini index and gain ratio were used to characterize purity and determine the optimal split node. A random forest (RF) is a collection of decision trees that individually act as weak classifiers, but overall form robust predictions. The RF was constructed by hyperparameter searching to optimize the number of trees and the number of leaf nodes in the forest to construct the optimal RF prediction model.

### 2.3 Cell culture and cell processing

The HGC‐27 and MKN‐45 cell lines were cultured in high‐glucose DMEM (Solarbio, Beijing, China) supplemented with 10% fetal calf serum (VivaCell, Shanghai, China). Both gastric cancer cell lines were cultured in an incubator at 37°C and 5% CO_2_. Gastric cancer cell lines were treated with the pathway inhibitor B02 (27.4 μM, MCE) for 24 h.

### 2.4 RNA extraction and qRT–PCR from cell lines and tissues

Total RNA was extracted using TransZol Up reagent (TransGen, China). The RNA was then reverse transcribed into cDNA using PrimeScript RT Reagent (TaKaRa, Japan), and the cDNA concentration was detected. GAPDH was used as the RNA internal reference, and the calculated Ct value was normalized to the GAPDH value. The difference was calculated using the −ΔCt method (ΔCt = Ct test − Ct GAPDH). The primer sequences used for PCR are listed in Table S1, available in Additional File 1, and were synthesized by Sangon Biotech (Shanghai, China).

### 2.5 Cell cycle analysis by flow cytometry

Gastric cancer cells were fixed overnight and then washed with PBS. The PI/RNase Staining cell cycle assay kit (DOJINDO, Japan) was used to prepare a working solution. The working solution was added to the cells, which were incubated for 30 min at 4°C in the dark. The cells were then incubated for 30 min at 37°C in darkness, and the proteins were detected by flow cytometry.

### 2.6 Cell apoptosis detection by flow cytometry

An apoptosis detection kit, Annexin V‐APC/PI (BestBio, China), was used to detect gastric cancer cells. In the first step, 5 μL of Annexin V‐APC stain was added to the cell suspension and incubated for 5–10 min in the dark. Then, 10 μL of PI stain was added, and the mixture was incubated for 1–3 min. Next, 400 μL of 1X Annexin V conjugate was added and mixed gently before flow cytometry.

### 2.7 Cytotoxicity assay

The treated gastric cancer cells were seeded in a 96‐well plate at a concentration of 1.5 × 10^3^/well and incubated for 24 h. Complete cisplatin culture medium (MCE, China) containing 0.5, 1, 2, 4, 8, 16, 0, 32, 64, or 128 μM/mL cisplatin was added sequentially to the 96‐well plates and incubated for 24 h. Then, SuperKine™ Ultrasensitive Cell Proliferation Detection Reagent (Abbkine, China) was added, the absorbance was measured at 450 nm after incubation for 2 h, and the half inhibitory concentration (IC50) curve was plotted.

### 2.8 Radiomics feature extraction and screening

CT images of gastric cancer in the venous stage were downloaded from two sources: the Imaging Centre of the First Affiliated Hospital of Nanchang University (training cohort) and The Cancer Genome Atlas database (https://www.cancerimagingarchive.net/) image database (external validation cohort). Tumor segmentation was performed manually by a radiologist with more than 5 years of experience in abdominal radiology using 3D Slicer software (version 5.61) and then checked by a surgeon with more than 20 years of experience in gastrointestinal surgery. The segmented image files were saved in Nrrd format, and features were extracted using the PyRadiomics plugin in 3D Slicer. According to the high and low expression of diagnostic biomarkers, the radiomics features were divided into high‐ and low‐expression groups; and feature screening was performed using LASSO regression. The radiomics scores of the biomarkers were calculated based on the correlation coefficients and constants of the screened features. The clinical relevance of the radiomics scores was further analyzed. For more details on the tumor segmentation method, feature extraction process, and types of radiomics features, please refer to our previous research.^25^

### 2.9 Statistical analysis

Machine learning screening of biomarkers was performed using R software (version 4.13) for statistical analysis; the support vector machine used the “e1071” package in R, and logistic regression used forwards‐backwards stepwise regression of the “rms” package in R. LASSO regression was used for feature selection using the “glmnet” package in R. For decision analysis, the “rpart” package in R was used for decision trees and random forest (RF). Differences in quantitative data were analyzed using the *t*‐test in GraphPad Prism 8 (version 8.0). Correlations between biomarkers, biomarker imaging scores, and clinicopathological features were analyzed using Spearman correlation analysis. *p* < 0.05 was considered to indicate statistical significance.

## 3 RESULTS

### 3.1 Screening of biomarkers for gastric cancer diagnosis

**FIGURE 2:**
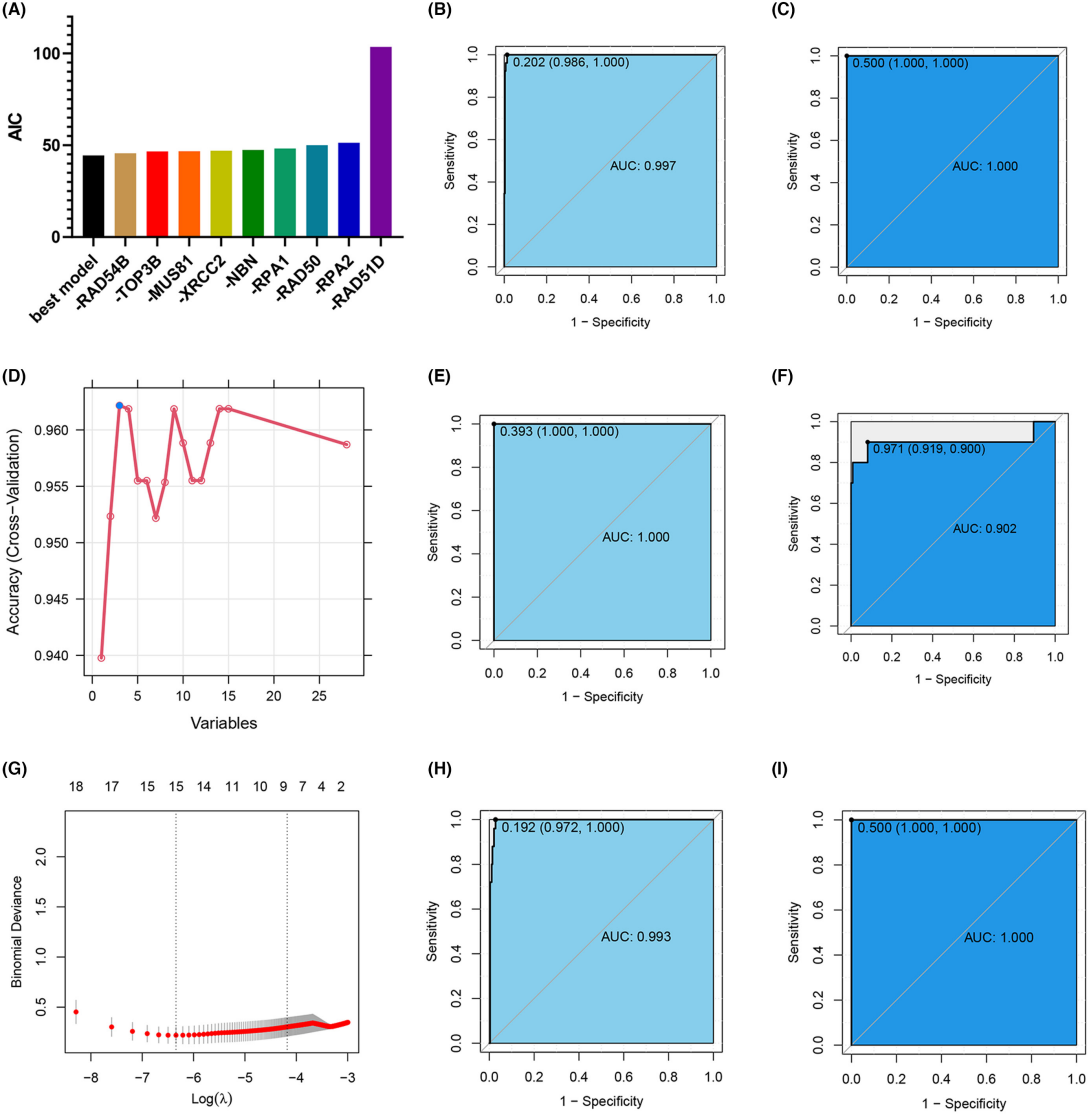
Logistic regression, LASSO regression, and SVM were employed to screen for gastric cancer biomarkers. Logistic regression‐screened biomarkers: (A) Predictive model AIC values; (B) ROC curve of the training cohort; (C) ROC curve of the test cohort. SVM‐screened biomarkers: (D) Relationship between the number of features and the model accuracy; (E) ROC curve of the training cohort; (F) ROC curve of the test cohort. LASSO regression‐screened biomarkers: (G) Relationship between the lambda values and bias percentage of features; (H) ROC curve of the training cohort; (I) ROC curve of the test cohort.

We screened homologous recombination genes using logistic regression, SVM, LASSO regression, DT, and random forest (RF) methods. According to logistic regression, when the number of feature genes was nine (Figure 2A), the smallest Akaike information criterion (AIC) was 44.43 was observed. Deletion of any of these genes increases the AIC, and RAD51D contributes the most to the AIC of the diagnostic prediction model for gastric cancer. The area under the curve (AUC) of the diagnostic prediction model was 0.997 in the training group and 1.000 in the testing group (Figure 2B,C), which indicates the high accuracy of the diagnostic biomarkers screened by logistic regression. The diagnostic prediction model constructed by SVM had the highest accuracy when the number of feature genes was three: RAD51D, BRCA2, and XRCC2 (Figure 2D). The AUC of the diagnostic prediction model in the training group was 1.000, versus 0.902 in the test group (Figure 2E,F), which still indicates a high degree of accuracy. In LASSO regression, as the lambda value continues to increase, the deviation percentage first decreases gradually and then increases gradually, and when the deviation percentage is the smallest, it corresponds to the best lambda value (Figure 2G). Nine feature genes were screened (Table S2), and the AUC of the diagnostic prediction model was 0.993 for the training group and 1.000 for the testing group (Figure 2H,I). The prediction model constructed by the SVM has very high accuracy in both the training and testing sets.

**FIGURE 3:**
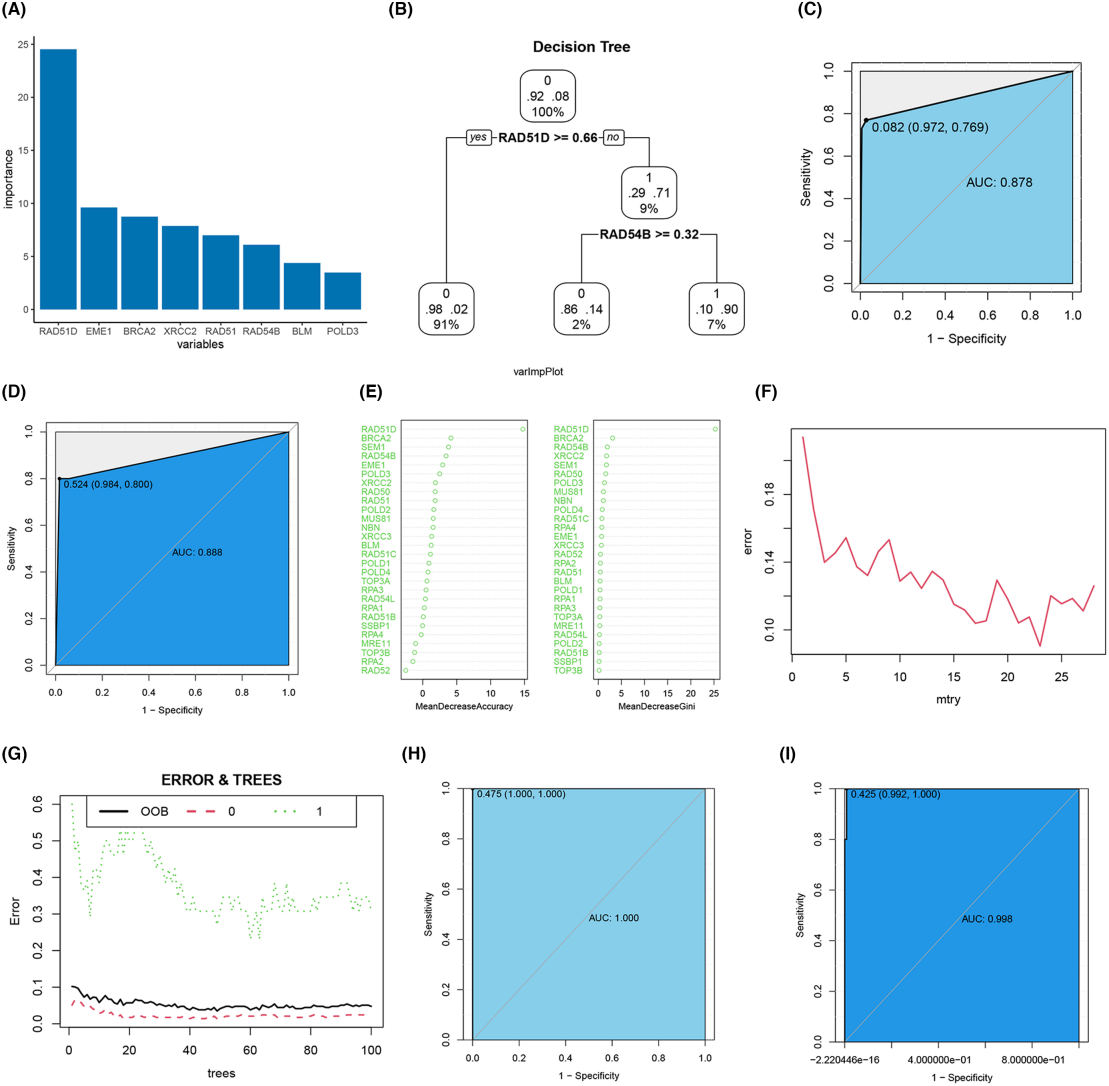
DT and RF were used to screen for gastric cancer biomarkers. DF‐screened biomarkers: (A) The significance of features for the predictive model; (B) Decision tree diagram; (C) The ROC curve of the training cohort; (D) The ROC curve of the test cohort. RF‐screened biomarkers: (E) Importance of features in improving the model accuracy and reducing the Gini coefficient; (F) Relationship between the number of split points and error in the tree; (G) Relationship between the number of trees and OOB error in RF, where “0” represents the normal group, and “1” represents the gastric cancer group; (H) The ROC curve of the training cohort; (I) The ROC curve of the test cohort.

Based on their importance, we ranked the homologous recombination genes in the decision tree used to construct the prediction model. The top 8 characteristic genes are shown in Figure 3A. Our analysis revealed that RAD51D significantly contributed to the construction of the decision tree prediction model. We have determined that the prediction model will have the highest accuracy when the number of split nodes of the decision tree is two (Figure 3B). We plotted the decision tree prediction model based on these findings. The AUC of the prediction model in the training cohort was 0.878, with a sensitivity of 0.769 and specificity of 0.972 (Figure 3C). The AUC of the prediction model in the test cohort was 0.888, with a sensitivity of 0.800 and specificity of 0.984 (Figure 3D). In the feature gene importance analysis of the random forest prediction model, RAD51D, BRCA2, and RAD54B among the six screened feature genes were found to play essential roles in the accuracy of the prediction model and in reducing the Gini coefficient of the prediction model (Figure 3E). When the number of splitting nodes of the tree is 23 (Figure 3F), the error is the smallest. When the number of trees in the prediction model is 40, the Out‐of‐Bag (OOB) error has a minimum value of 0.381 (Figure 3G). The AUC of the prediction model in the training cohort was 1.000, and the AUC in the test cohort was 0.998 (Figure 3H,I). The gastric cancer diagnosis prediction model constructed by the random forest algorithm had very high accuracy for both the training and test sets.

### 3.2 Differential expression and prognosis of RAD51D and XRCC2

**FIGURE 4:**
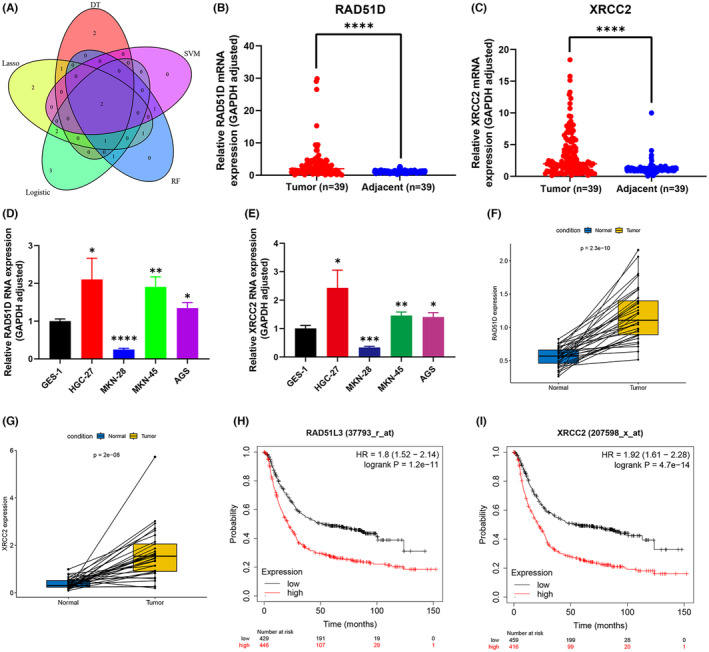
(A) Venn diagram; (B, C) Differential expression of RAD51D and XRCC2 in 39 pairs of gastric cancer tissues and adjacent tissues; (D, E) Differential expression of RAD51D and XRCC2 in gastric cancer cell lines; (F, G) Differential expression of RAD51D and XRCC2 in gastric cancer tissues and paired normal tissues; (H, I) Relationship between the expression of RAD51D and XRCC2 and prognosis.

We used the intersection of diagnostic biomarkers screened by machine learning and obtained RAD51D and XRCC2 (Figure 4A). The mRNA levels of RAD51D and XRCC2 in gastric cancer tissues were significantly greater than those in adjacent tissues (*p* < 0.001) (Figure 4B,C). In the gastric cancer cell lines HGC‐27, MKN‐45, and AGS, the mRNA levels were also considerably greater than those in the normal gastric mucosal cell line GES‐1 (Figure 4D,E). In addition, we used the TCGA database to verify that after pairing RAD51D and XRCC2, the mRNA level in gastric cancer tissue was still significantly greater than that in adjacent tissue (*p* < 0.001) (Figure 4F,G). High expression of RAD51D and XRCC2 was associated with poor prognosis in gastric cancer patients according to survival analysis conducted on the Kaplan–Meier Plotter website (http://kmplot.com/analysis/) (Figure 4H,I). The above results indicate that RAD51D and XRCC2 play crucial roles in the diagnosis and prognosis of gastric cancer.

### 3.3 Correlations of RAD51D and XRCC2 with clinicopathological features

**FIGURE 5:**
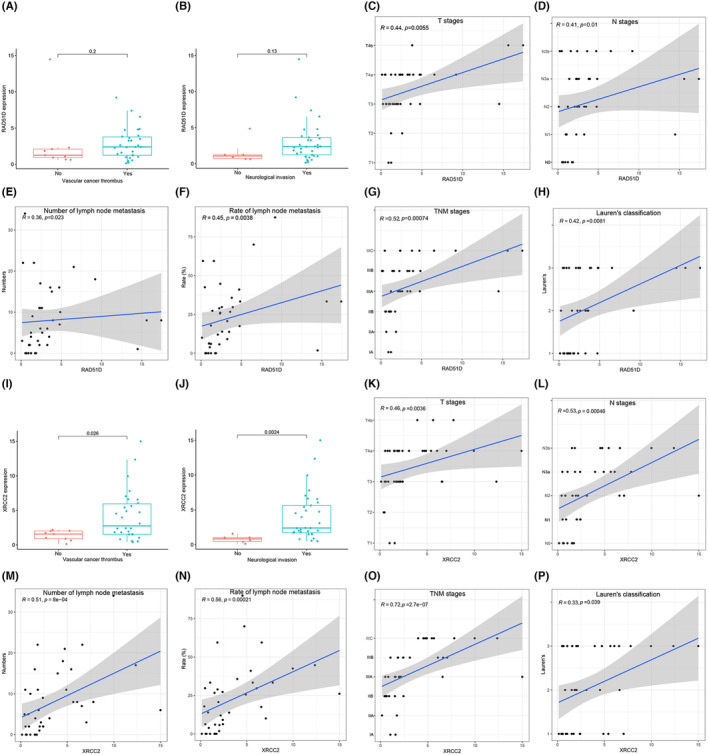
(A–H) Correlations of RAD51D with vascular cancer thrombus status, neurological invasion status, pathological T stage, N stage, number of lymph node metastases, lymph node metastasis rate, TNM stage, and Lauren's classification. (I–P) Correlation of XRCC2 with vascular cancer thrombus, neurological invasion, pathological T stage, N stage, number of lymph node metastases, lymph node metastasis rate, TNM stage, and Lauren's classification.

In the previous results, qRT‐PCR assays measured the expression of RAD51D with XRCC2 in 39 gastric cancer patients. We combined the mRNA levels of RAD51D and XRCC2 with the pathological features of the corresponding patients. RAD51D expression was not significantly correlated with vascular thrombus or nerve invasion (Figure 5A,B), while XRCC2 expression was greater in gastric cancer patients with vascular thrombus or nerve invasion (*p* < 0.05) (Figure 5I,J). The expression levels of RAD51D and XRCC2 were significantly correlated with pathological T stage, N stage, number of lymph node metastases, lymph node metastasis rate, TNM stage, and Lauren's classification (*p* < 0.05) (Figure 5C–H,K–P). On the other hand, there was no significant correlation between the expression levels of RAD51D or XRCC2 and lymphatic vessel invasion, vascular invasion, tumor size, p53 expression, CerbB2 expression, or Ki‐67 expression (Figure S1). Furthermore, we performed a correlation analysis of RAD51D and XRCC2 expression with pathological features in the TCGA database. The results revealed that there was a significant positive correlation between the expression of RAD51D and XRCC2 and the pathological T stage of gastric cancer (Figure S2).

### 3.4 Biological functions of the pathways associated with RAD51D and XRCC2

**FIGURE 6–:**
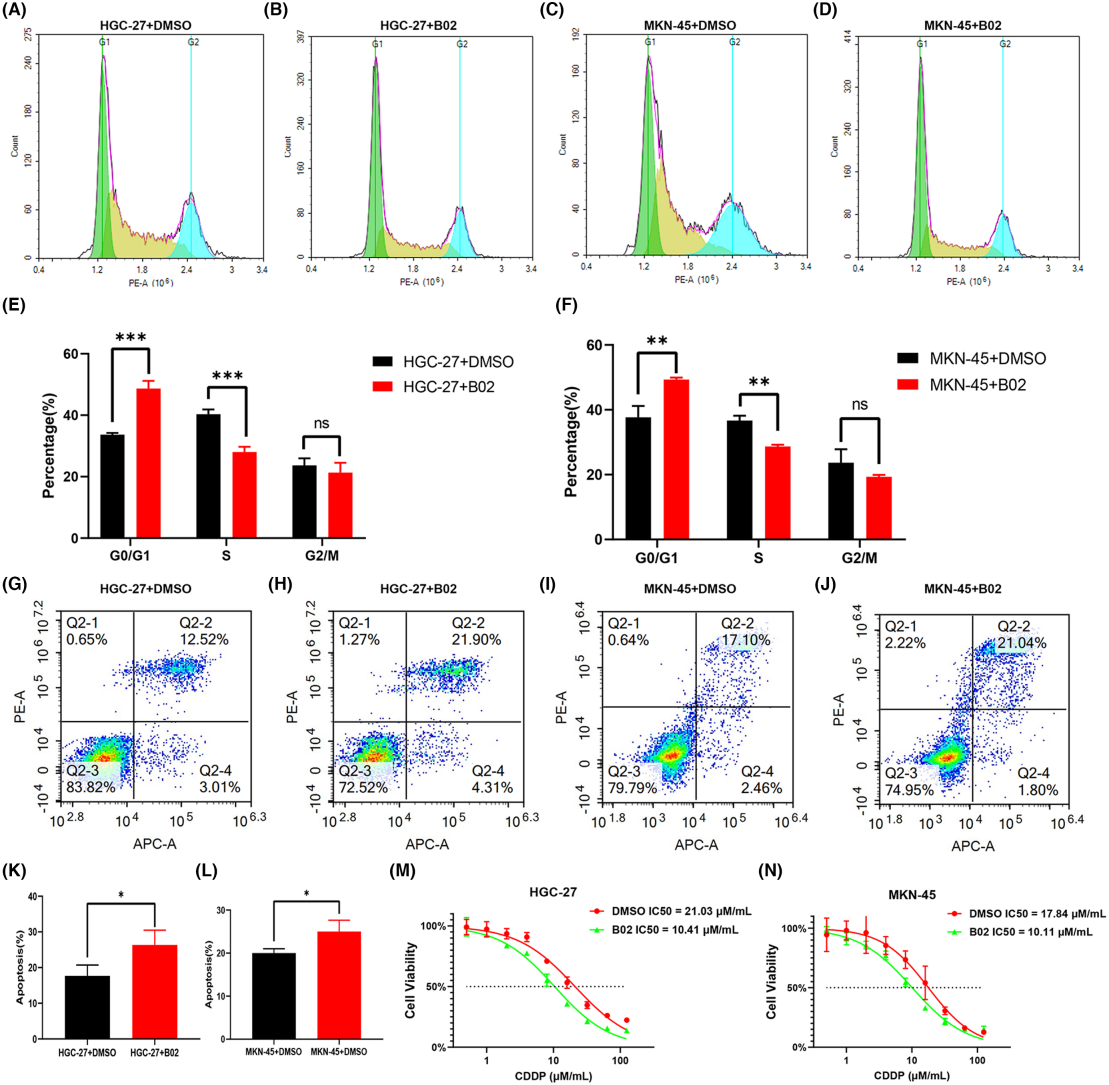
HGC‐27 cell line: (A, B) Flow cell cycle diagram of treated and control groups; (E) Comparison of cell cycle differences between treated and control groups; (G, H) Flow cell apoptosis diagram of treated and control groups; (K) Comparison of apoptosis percentage differences between treated and control groups; (M) IC50 curve of the HGC‐27 cell line. MKN‐45 cell line: (C, D) Flow cell cycle diagram of the treated and control groups; (F) Comparison of cell cycle differences between the treated and control groups; (I, J) Flow cell apoptosis diagram of the treated and control groups; (L) Comparison of apoptosis percentage differences between the treated and control groups; (N) IC50 curve of the MKN‐45 cell line.

Since the main role of the homologous recombination pathway is to participate in DNA damage repair, we used the pathway inhibitor B02 to treat HGC‐27 and MKN‐45 cells to observe its effects on the cell cycle, apoptosis, and sensitivity to chemotherapy drugs. The results showed that after the B02 treatment, the proportion of HGC‐27 cells in the S phase was significantly lower than that in the control group, and the proportion of HGC‐27 cells in the G0/G1 phase was significantly greater than that in the control group. The difference was statistically significant (Figure 6A,B,E). The results observed in the MKN‐45 cell line were similar to those in the HGC‐27 cell line after B02 treatment (Figure 6C,D,F). On the other hand, the proportion of apoptotic HGC‐27 cells after B02 treatment reached 26%, and the proportion of apoptotic HGC‐27 cells in the control group treated with DMSO was 15% (Figure 6G,H,K). The difference was statistically significant (*p* < 0.05). The apoptosis results of the MKN‐45 cell line after B02 treatment were similar to those of the HGC‐27 cell line (Figure 6I,J,L). As the first‐line chemotherapy drug for gastric cancer treatment, we chose to administer cisplatin to verify the sensitivity of tumor cells to chemotherapy. The IC50 value of the HGC‐27 cell line after B02 treatment was 10.41 μM/mL, and the IC50 value of the control group was 21.03 μM/mL (Figure 6M). The difference was statistically significant (*p* < 0.05). The MKN‐45 cell line showed similar results as the HGC‐27 cell line: the homologous inhibitor B02 increased the sensitivity of gastric cancer cells to chemotherapy drugs (Figure 6N). The above results show that blocking the homologous recombination pathway inhibits the proliferation of gastric cancer cells, promotes the apoptosis of gastric cancer cells, and reduces the sensitivity of gastric cancer cells to chemotherapy drugs.

### 3.5 Construction and validation of the RAD51D and XRCC2 radiomics prediction models

**FIGURE 7:**
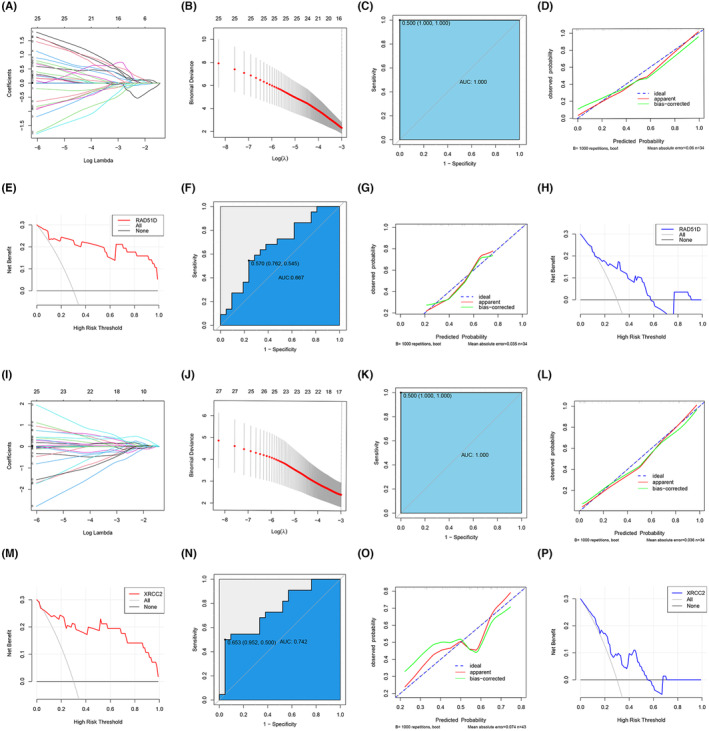
(A–H) Construction and evaluation of the radiomics model for predicting RAD51D expression. (A) Relationships between the lambda values and radiomics feature coefficients; (B) Relationships between the lambda values and bias percentages of radiomics features; (C) ROC curve of the training cohort; (D) Calibration curve of the predictive model in the training cohort. The abscissa represents the predictive probability of RAD51D expression status, and the ordinate represents the actual probability of RAD51D expression status; (E) DCA of the training cohort, where “All” indicates that all patients were treated with cisplatin‐containing chemotherapy regimens and “None” indicates that all patients were treated with cisplatin‐free chemotherapy regimens; (F) ROC curve of the validation cohort; (G) Calibration curve in the validation cohort; (H) DCA of the validation cohort; (I–P) Construction and evaluation of the radiomics model for predicting XRCC2 expression.

Our previous results showed that inhibition of the homologous recombination pathway significantly impacts patient sensitivity to platinum‐based chemotherapy drugs. Analysis through the ChIPBase v3.0 online website (https://rnasysu.com/chipbase3/index.php) revealed that the expression of RAD51D and XRCC2 was significantly positively correlated with that of RAD51, an essential gene for homologous recombination (Figure S3). Therefore, we could predict the expression status of RAD51D and XRCC2 during homologous recombination to reflect the sensitivity of gastric cancer patients to platinum chemotherapy drugs. Through feature extraction, we extracted 864 radiomics features (Table S3). Due to the large number of radiomics features, we further used LASSO regression for feature screening. Through this process, we identified 17 radiomics features related to high and low expression of RAD51D and 18 radiomics features associated with high and low expression of XRCC2 (Figure 7A,B,I,J). The screened radiomics features were then used to construct prediction models for high and low expression of RAD51D and XRCC2 through logistic regression. In the model predicting RAD51D expression, the AUC of the training group was 1.000 (Figure 7C); the calibration curve showed that the actual prediction curve did not deviate from the ideal curve; the C‐index of the prediction model was 0.94; and the calibration C‐index was 0.89, which indicated high accuracy of the prediction model (Figure 7D). The decision curve analysis (DCA) showed that the clinical benefit of selecting a chemotherapy regimen by predicting RAD51D expression status was significantly greater than that of chemotherapy regimens that only used cisplatin or did not use cisplatin (Figure 7E). In the external validation group, the AUC of the prediction model was 0.667, the sensitivity was 0.762, and the specificity was 0.545 (Figure 7F); the calibration curve showed that the actual prediction curve was still near the ideal curve, the C‐index of the prediction model was 0.60, and the calibration C‐index was 0.56 (Figure 7G); the DCA showed that predicting RAD51D expression status to select chemotherapy regimens still confers good clinical benefit (Figure 7H).

In the model for predicting XRCC2 expression, the AUC of the training cohort was 1.000 (Figure 7K), the prediction curve was close to the ideal curve, the C‐index of the prediction model was 0.94, and the calibration C‐index was 0.92 (Figure 7L). The DCA showed that the chemotherapy regimen selected for predicting the expression status of XRCC2 had a significant clinical benefit (Figure 7M). In the external validation cohort, the AUC of the prediction model was 0.742, the sensitivity was 0.952, and the specificity was 0.500 (Figure 7N). Although the prediction curve had certain fluctuations, it still surrounded the ideal curve (Figure 7O). The C‐index of the prediction model was 0.74, and the calibration C‐index was 0.63; the DCA showed that predicting the expression status of XRCC2 to select chemotherapy regimens also conferred good clinical benefit (Figure 7P). The above results show that CT radiomics features can reflect the expression status of RAD51D and XRCC2 to a certain extent and have good accuracy in the external validation cohort.

### 3.6 Clinical correlation analysis of RAD51D and XRCC2 radiomics scores

**FIGURE 8:**
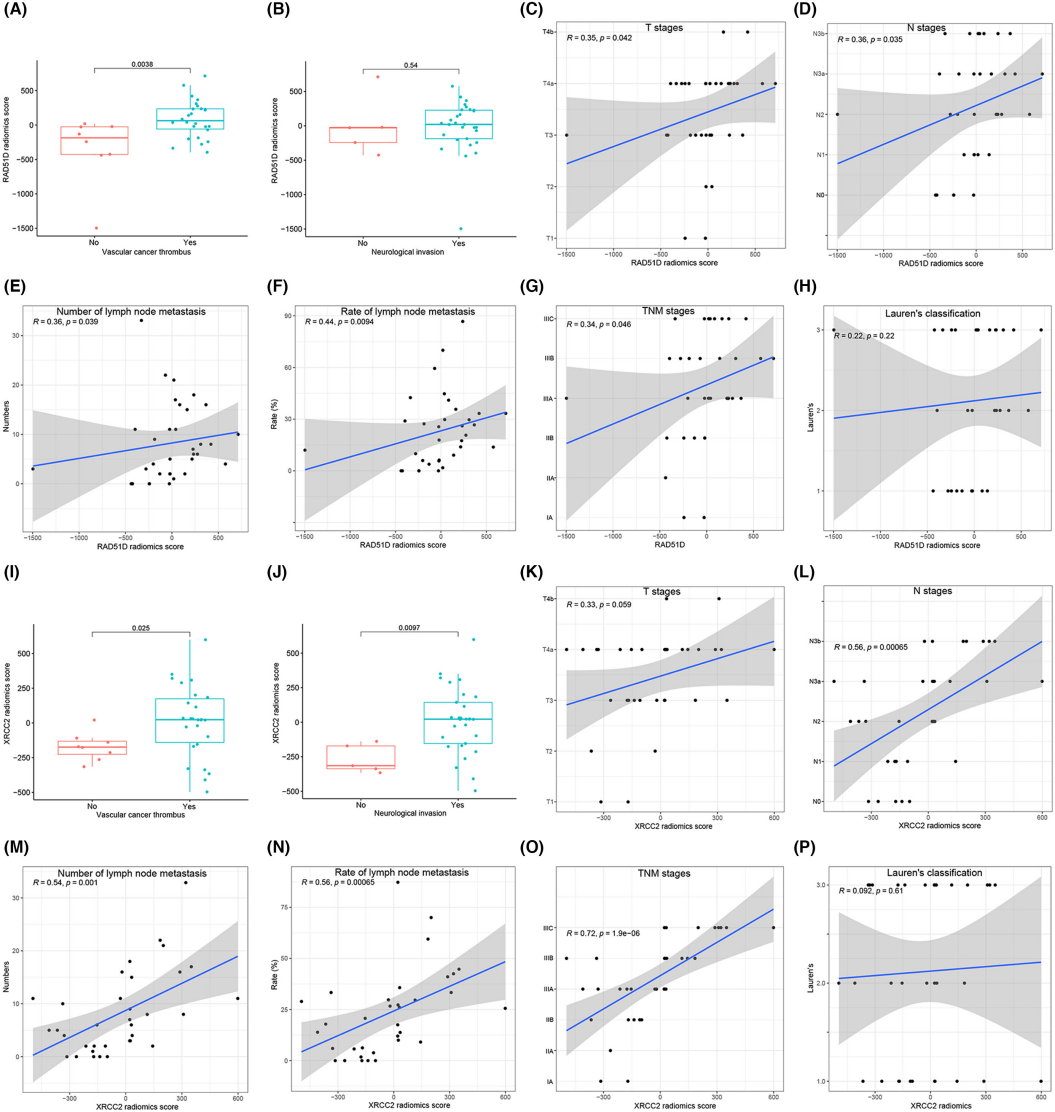
(A–H) Correlations of RAD51D radiomics score with vascular cancer thrombus status, neurological invasion status, pathological T stage, N stage, number of lymph node metastases, lymph node metastasis rate, TNM stage, and Lauren's classification. (I–P) Correlation of XRCC2 radiomics scores with vascular cancer thrombus, neurological invasion, pathological T stage, N stage, number of lymph node metastases, lymph node metastasis rate, TNM stage, and Lauren's classification.

Using the predictive RAD51D and XRCC2 expression model constructed by logistic regression, we calculated the radiomics scores of RAD51D and XRCC2. The radiomics score calculation formula for RAD51D was as follows: radiomics score = −14.02 + OSMD × 272.55 + OGIV × −157.2 + WLGCP × −88.23 + WLFOMAD × 373.31 + WLGL × 509.57 + WHGSAE × −163.56. The radiomics score calculation formula for XRCC2 was as follows: radiomics score = −31.64 + DIOM × −95.82 + ONS × −80.4 + WLFS × −230.57 + WLNB × 53.5 + WHNC × −137.36. The results showed that the radiomics score was greater in the group with vascular tumor thrombus and was not significantly correlated with nerve invasion (Figure 8A,B). In comparison, the XRCC2 radiomics score was greater in gastric cancer patients with vascular tumor thrombus or nerve invasion (*p* < 0.05) (Figure 8I,J). The RAD51D radiomics score was significantly positively correlated with pathological T stage, N stage, number of lymph node metastases, lymph node metastasis rate, and TNM stage (*p* < 0.05) but was not significantly correlated with Lauren's classification (Figure 8C–H). The XRCC2 radiomics score was significantly positively correlated with the N stage, number of lymph node metastases, lymph node metastasis rate, and TNM stage (*p* < 0.05) but was not significantly correlated with the T stage or Lauren's classification (Figure 8K–P). There was no significant correlation between RAD51D or XRCC2 radiomics score and lymphatic vessel invasion, vascular invasion, tumor size, p53 expression level, CerbB2 expression level, or Ki‐67 expression level (Figure S4).

## 4 DISCUSSION

In recent years, homologous recombination has been increasingly studied in tumors. Research on gastric cancer has focused on two main aspects. On the one hand, homologous recombination serves as a risk factor for gastric cancer and promotes its occurrence. Patients with *H. pylori* infection and specific homologous recombination gene mutations face a significantly greater risk of developing gastric cancer. On the other hand, homologous recombination plays a crucial role in promoting DNA damage repair. When abnormalities occur in the homologous recombination pathway, they can affect the sensitivity of gastric cancer patients to chemotherapy. To this end, our research primarily focuses on these two aspects. First, we screened homologous recombination genes as diagnostic biomarkers for gastric cancer. Then, we used CT radiomics to construct a predictive model based on these diagnostic biomarkers to predict the sensitivity of gastric cancer patients to chemotherapy. This model can guide the selection of chemotherapy regimens for gastric cancer patients in the clinic.

We used LASSO regression, logistic regression, support vector machine, decision tree, and random forest methods to screen for diagnostic biomarkers of gastric cancer. Each machine learning feature screening principle is different, and the features that are screened out vary greatly. In addition, various machine learning methods have been widely used in gastric cancer, and they all achieve high accuracy. Our choice of which machine learning feature should be used as the criterion is challenging.^26^, ^27^, ^28^, ^29^, ^30^ To this end, we intersected the gastric cancer diagnostic biomarkers screened by each machine learning method and obtained RAD51D and XRCC2. Strikingly, regardless of which kind of gastric cancer prediction model is built by machine learning, RAD51D has the most prominent contribution to the accuracy of the prediction model. RAD51D is one of five homologs of RAD51, including RAD51B, RAD51C, XRCC2, and XRCC3.^31^ RAD51 is a crucial enzyme for homologous recombination and mainly plays a role in DNA damage repair. RAD51D, as a cofactor in the homologous recombination pathway, plays an indispensable role in cooperation with RAD51.^32^ Many studies have confirmed that RAD51C and RAD51D germline pathogenic variants are closely associated with the development of ovarian and breast cancer.^33^, ^34^ A study from Japan evaluated the relationship between germline pathogenic variants in 27 cancer susceptibility genes and the risk of gastric cancer. The results showed that RAD51D was not a risk factor for gastric cancer, but other BRCA1, BRCA2, and ATM genes from the homologous recombination pathway are risk factors for gastric cancer.^9^ Our study identified RAD51D as a diagnostic biomarker for gastric cancer and validated it in gastric cancer tissues and cell lines. At present, research on RAD51D has mainly assessed the relationship between germline pathogenic variants and cancer risk, and there is a lack of research on the specific molecular mechanisms by which RAD51D causes cancer.

In this study, we performed correlation analyses of RAD51D and XRCC2 expression with pathological features rather than difference analyses with pathological features. We did not perform a difference analysis due to the limited number of patients included. If a difference analysis of the expression of RAD51D and XRCC2 with T stage, N stage, and TNM stage had been performed, the number of patients assigned to each group would have been small. The small number of patients can lead to negative results for pathological features that were originally positive. To verify the relationships between RAD51D and XRCC2 radiomics scores and pathological features, we also performed correlation analysis because of the small number of patients.

Radiomics converts specific images into quantitative feature data that can be analyzed and has been widely used for research in gastric cancer, mainly in the staging of lymph node metastasis, postoperative survival time, complete pathological remission after neoadjuvant therapy, and prediction of immunotherapeutic sensitivity.^35^, ^36^, ^37^, ^38^ Research reflecting the microscopic heterogeneity of tumors through radiomics has developed rapidly in recent years.^39^ For example, the prognostic radiomics score for advanced breast cancer shows significant differences in GSEA enrichment, tumor immune phenotype, and immune cell type among patients with advanced breast cancer.^40^, ^41^ Prognostic radiomics features in glioblastoma multiforme screening are associated with immune cell infiltration and may predict the clinical response to immunotherapy.^42^ Similarly, the low‐ and high‐radiomics score groups of hepatocellular carcinoma can display different tumor microenvironments.^43^ However, no studies have been conducted on the correlation between radiomics features and cancer genes. Our study predicted high and low expression of RAD51D and XRCC2 through radiomics features. In the training cohort, the AUC values of RAD51D and XRCC2 in the prediction model were almost 1.000. Although we did not have an internal validation group, it did not affect the results of the prediction model construction in the training group or the external validation group. In the external validation cohort, the AUC of the RAD51D prediction model was 0.667, and the AUC of the XRCC2 prediction model was 0.742. In this external validation group, the expression prediction models of RAD51D and XRCC2 still had excellent AUC values. Radiomics features can reflect the expression status of cancer genes to a certain extent. We also believe that more studies on radiomics features reflecting the expression status of cancer genes will be published shortly.

Using machine learning, we screened RAD51D and XRCC2 in the homologous recombination pathway as diagnostic biomarkers for gastric cancer. Moreover, our study showed that the expression of RAD51D and XRCC2 was significantly and positively correlated with the clinicopathological features of gastric cancer patients. In addition, our study showed that inhibiting the homologous recombination pathway could improve the sensitivity of gastric cancer patients to platinum‐based chemotherapeutic agents. We constructed a radiomics model to predict the expression of RAD51D and XRCC2. This model also showed specific predictive performance in the external validation cohort, indicating that CT radiomics can, to a certain extent, reflect the expression of RAD51D and XRCC2. This can help identify the possibility of gastric cancer patients being resistant to platinum chemotherapy and guide the selection of chemotherapy regimens for gastric cancer patients. However, our research has specific areas for improvement. The number of patients in the training group we used to construct the RAD51D and XRCC2 expression status prediction model was limited due to the small number of patients who had both RAD51D and XRCC2 expression data and radiomics data. Therefore, our results need to be verified by studies with larger sample sizes.

## AUTHOR CONTRIBUTIONS

**Ahao Wu:** Conceptualization (equal); writing – original draft (equal). **Tengcheng Hu:** Methodology (equal). **Chao Lai:** Writing – original draft (equal). **Qingwen Zeng:** Data curation (equal). **Lianghua Luo:** Formal analysis (equal). **Xufeng Shu:** Methodology (equal). **Pan Huang:** Methodology (equal); resources (equal). **Zhonghao Wang:** Formal analysis (equal); supervision (equal). **Zongfeng Feng:** Formal analysis (equal); supervision (equal). **Yanyan Zhu:** Software (lead). **Yi Cao:** Writing – review and editing (equal). **Zhengrong Li:** Funding acquisition (lead); project administration (equal); writing – review and editing (lead).

## FUNDING INFORMATION

This work was supported by the leading scientist Project of the Jiangxi Science and Technology Department (20213BCJL22050), Jiangxi Provincial Natural Science Foundation (20224ACB206029), the National Natural Science Foundation of China (82360525), and Jiangxi Provincial Natural Science Foundation (20224BAB206063).

## CONFLICT OF INTEREST STATEMENT

All authors declare that the research was conducted in the absence of any commercial or financial relationships.

## ETHICS STATEMENT

This study was approved by the Ethics Committee of the First Affiliated Hospital of Nanchang University [Ethics Committee Approval No: (2023) CDYFYYLK (09‐009)]. All patients signed informed consent for this study. All methods were performed according to relevant guidelines and regulations.

## Supporting information


Data S1.

Data S2.

## Data Availability

The datasets used and/or analyzed during the current study are available from the corresponding author upon reasonable request.
