# Supplementary material for: Screening of gastric cancer diagnostic biomarkers in the homologous recombination signaling pathway and assessment of their clinical and radiomic correlations
Source: Cancer Med. 2024 Aug 29;13(16):e70153. doi: 10.1002/cam4.70153 (PMC11358765; doi:10.1002/cam4.70153)
Supplement: Supplementary file 1 — Data S1. [file CAM4-13-e70153-s002.docx]

Supplementary table S1. Primer sequences for GAPDH, RAD51D, and XRCC2

| Gene | Primer Sequences |
| --- | --- |
| GAPDH_F | CCCACTCCTCCACCTTTGAC |
| GAPDH_R | CCACCACCCTGTTGCTGTAG |
| RAD51D_F | CTTTGTGCCCAGCACTCGGATT |
| RAD51D_R | GTCTACCATCTCCTGGAAACCTG |
| XRCC2_F | TCTGTTTGCTGATGAAGATTCACC |
| XRCC2_R | CATCGTGCTGTTAGGTGATAAAGC |

Supplementary table S2. Biomarkers screened by machine learning.

| Machine Learning Methods | Biomarkers |
| --- | --- |
| Logistic Regression | NBN, RAD51D, XRCC2, RPA1, RAD50, RPA2, MUS81, RAD54B, TOP3B |
| Lasso Regression | RAD54L, NBN, RAD51D, XRCC2, RAD51, POLD3, MUS81, RAD54B, SEM1 |
| Support Vector Machine | RAD51D, BRCA2, XRCC2 |
| Decision Tree | RAD51D, DEME1, RCA2, XRCC2, RAD51, RAD54B |
| Random Forest | RAD51D, BRCA2, RAD54B, XRCC2, SEM1, RAD50 |


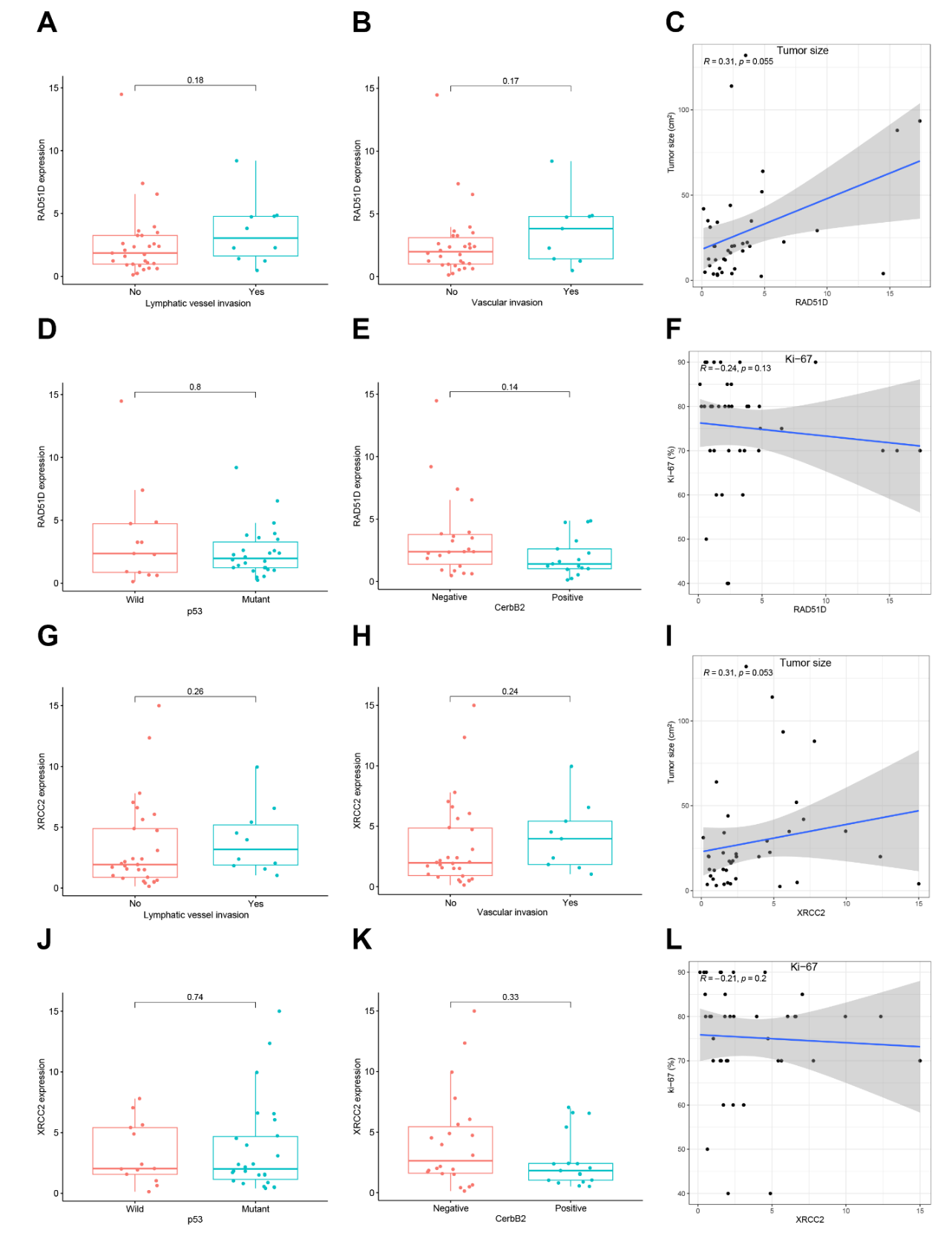
 Supplementary Fig. S1: (A-F) Correlation of RAD51D expression with lymphatic vessel invasion, vascular invasion, tumor size, p53, CerbB2, and Ki-67; (G-L) correlation of XRCC2 expression with lymphatic vessel invasion, vascular invasion, tumor size, p53, CerbB2, and Ki-67.

Supplementary Fig. S2: (A-C) Correlation of RAD51D expression with T stage, N stage, and TNM stage; (D-F) Correlation of XRCC2 expression with T stage, N stage, and TNM stage.


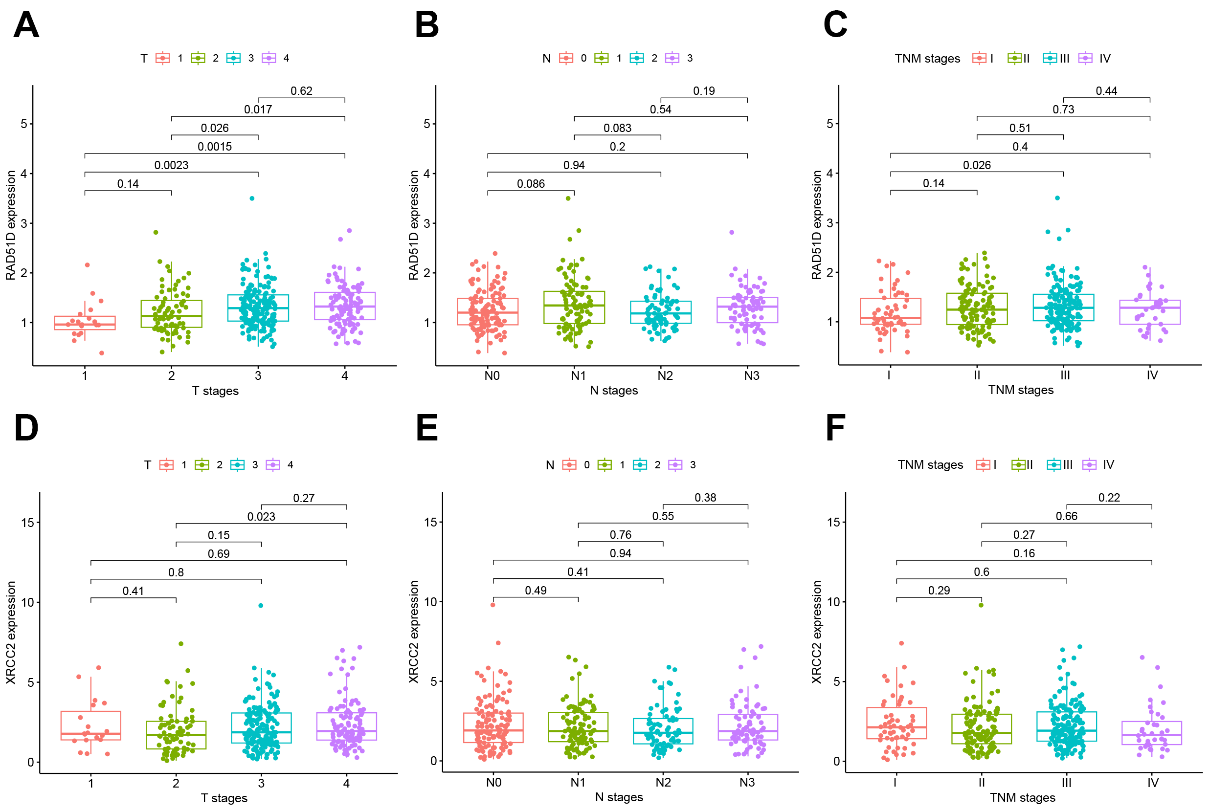


Supplementary
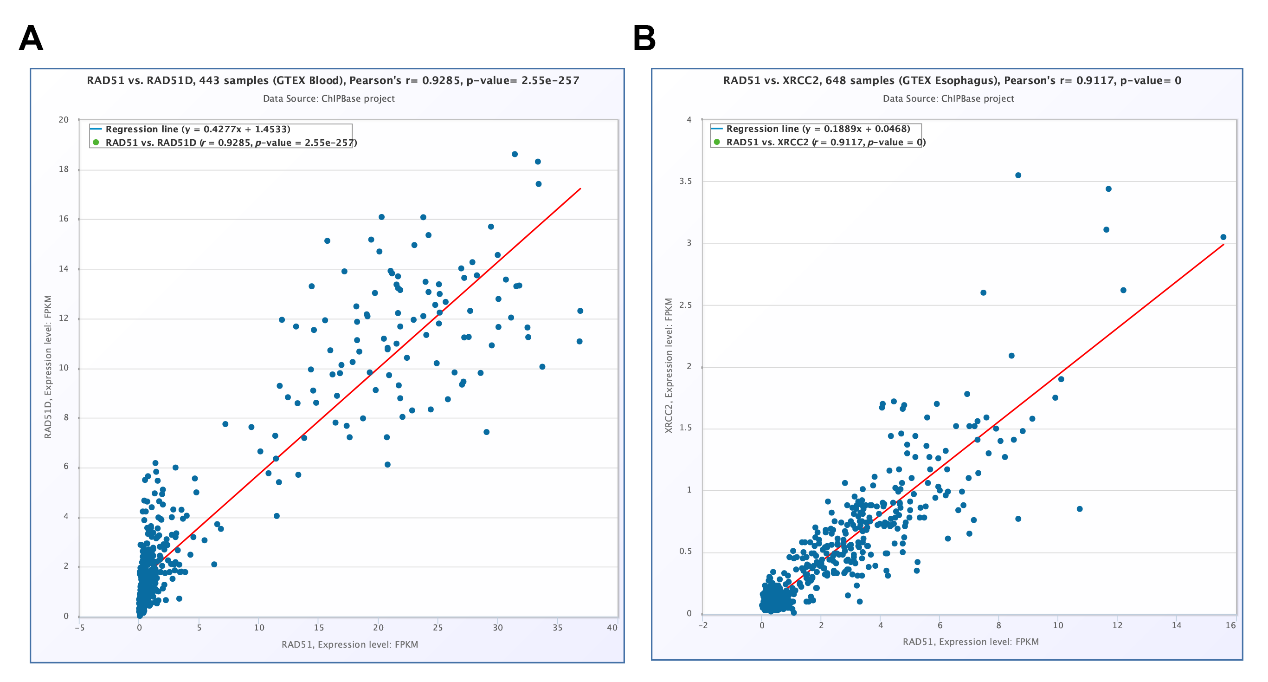
Fig. S3: (A) Correlation of RAD51D with RAD51; (B) correlation of XRCC2 with RAD51.

Supplementary
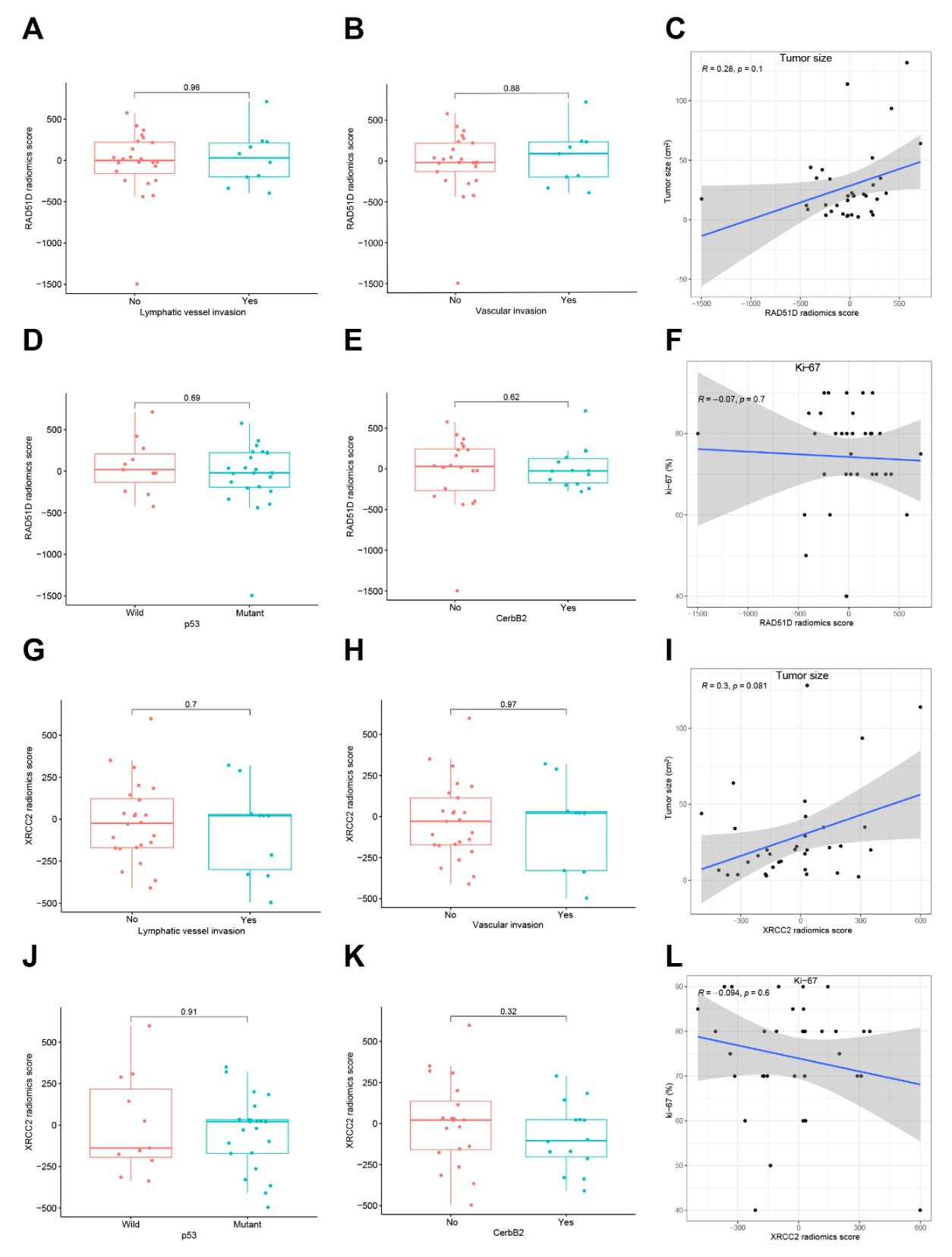
Fig. S4: (A-F) Correlations of RAD51D radiomics scores with lymphatic vessel invasion, vascular invasion, tumor size, p53, CerbB2, and Ki-67; (G-L) correlation of XRCC2 radiomics scores with lymphatic vessel invasion, vascular invasion, tumor size, p53, CerbB2, and Ki-67.
